# Antioxidant Capacity and Behavioral Relevance of a Polyphenolic Extract of *Chrysanthellum americanum* in a Rat Model of Irritable Bowel Syndrome

**DOI:** 10.1155/2019/3492767

**Published:** 2019-08-14

**Authors:** Roxana Cojocariu, Alin Ciobica, Ioana-Miruna Balmus, Samson Guenne, Anca Trifan, Carol Stanciu, Luminita Hrițcu, Radu Lefter

**Affiliations:** ^1^Department of Biology, Faculty of Biology, Alexandru Ioan Cuza University, 11th Carol I Avenue, 700506 Iasi, Romania; ^2^Department of Research, Faculty of Biology, Alexandru Ioan Cuza University, 11th Carol I Avenue, 700506 Iasi, Romania; ^3^Laboratory of Applied Biochemistry and Chemistry, University Joseph KI-ZERBO, 03 BP 7021 Ouagadougou 03, Burkina Faso; ^4^“Grigore T. Popa” University of Medicine and Pharmacy, 16th Universitatii Street, 700115 Iasi, Romania; ^5^Center of Biomedical Research, Romanian Academy, 8th Carol I Avenue, 700506 Iasi, Romania; ^6^Faculty of Veterinary Medicine, University of Agricultural Sciencies and Veterinary Medicine “”Ion Ionescu de la Brad” of Iasi, 3rd Mihail Sadoveanu Alley, Iasi 700490, Romania

## Abstract

*Chrysanthellum americanum* L. (Vatke) is a medicinal plant from the *Compositae* family used in west-African traditional medicine, known for its flavonoid and saponin richness and for its strong antioxidant potential. In the present study, we assessed the effects of *Chrysanthellum americanum* polyphenolic extract in the psychological stress-induced rat model of irritable bowel syndrome (IBS), a chronic functional digestive tract disorder marked by immune and inflammatory-related disturbances of central nervous and peripheral intestinal systems, which is often associated with mood disorders including depression and anxiety. Consequently, memory impairment, anxiety and depression behavioral indicators, and cerebral oxidative stress biomarker dynamics were evaluated in a multifactorial heterotypic stress-exposed IBS rats after 6-day gavage with polyphenolic *C. americanum* extract (100 mg/kg body weight). Y-maze, elevated plus maze, and forced swimming tests were used for assessing behavioral responses. Administration of the extract exhibited significant anxiolytic and antidepressant-like effects coupled with significantly increased temporal lobe antioxidant enzyme specific activity (superoxide dismutase and glutathione peroxidase) and decreased malondialdehyde levels, a well-known lipid peroxidation marker. Furthermore, linear regression statistical analyses showed significant correlations between the oxidative stress parameters and behavioral tests. In conclusion, our results suggest that the administration of *Chrysanthellum americanum* polyphenolic extract could ameliorate mood and cognitive disturbances related to stress-induced in an IBS rat model. This could be also related to cerebral oxidative stress status attenuation.

## 1. Introduction

Irritable bowel syndrome (IBS) is a functional digestive disorder characterized by abdominal pain, bloating, and stool irregularities marked by diarrhea, constipation, or recurrent passage from one state to another [1], without any apparent abnormal structural lesions or biochemical modifications. The worldwide prevalence of IBS in adults as estimated by several meta-analyses was reported to be around 10-20%, although varying largely from country to country [2], with 1.5 higher odds ratio for women [3].

When considering pathogenesis of IBS, heterogeneity is the main feature of the multiple triggering factors contributing to the characteristic symptoms: genetic susceptibility, acute gastrointestinal infections, gut microbiota alterations, activation of the gut immune system, inflammatory changes, dysregulation of the brain-gut axis, abnormal gut motor, and sensory functioning [4–7]. Psychological stress represents also a major factor in IBS symptom occurrence, as the dysregulation of the hypothalamic-pituitary-adrenal- (HPA-) axis response under excessive or prolonged stress induces visceral hyperalgesia, altered colonic motility, and intestinal transit in both healthy humans and animals [8]. Interactions between immune system and stress response appear to be one of the underlying mechanisms in the IBS outbreaks [9, 10], as shown in some studies highlighting activation of intestinal mucosal mast cells and other inflammatory mediators under chronic stress [11, 12], while all the more low-grade inflammation or immune activation (e.g., increase in the number of mast cells and lymphocytes, cytokine-related alterations) is more frequently reported in IBS patients than in healthy controls [13]. The corticotropin-releasing factor (CRF) family of peptides seems to be involved in the activation of inflammatory responses: urocortin was found to significantly induce the activation and degranulation of rat lung mast cells through CRF-R1 receptors with subsequent rapid release of inflammatory mediators [10, 14].

Similarly, it was shown that stress exposure could lead to gut microbiota alterations. In this way, beneficial probiotic bacteria such as *Lactobacillus* sp. reduction and gram-negative pathogenic bacteria such as *Escherichia coli* and *Pseudomonas* overgrowth, epithelial adherence, and mucosal uptake were well documented [15, 16]. Thus, gut microbiota dysbiosis could play an important role in IBS pathogenesis [15]. An experimental study found exaggerated HPA stress response in germ-free mice vs. gnotobiotic mice, which was reversed by reconstitution with *Bifidobacterium infantis*, while in contrast, monoassociation with enteropathogenic *Escherichia coli* enhanced the response to stress [17].

Although a certain predominant cause may stand out, in the pathophysiology of IBS, subsequent interplay of codependent factors would lead to manifestation of similar symptomtology [18]. Psychological stress seems to play a pivotal role in these interactions including activation of the mucosal immune system, disturbance of intestinal function (not uncommonly) accompanied by inflammatory process, and dysbiosis by impacting the pathways on the brain-gut axis (e.g., activating the HPA axis, via the vague nerve, various neuroendocrine agents) that link the “central nervous system, peripheral neurons, and gastrointestinal microbiota” [9, 19]. Disturbance of the brain-gut axis is encountered in other psychiatric disorders, such as the mood disorders, anxiety, depression [20, 21], and autism [22]; furthermore, mood disorders, which are stress-related disorders, are comorbid at least in 40-60% IBS patients [19]. These facts constitute the rationale for the current use of stress exposure, under a variety of approaches (neonatal and early life stress, chronic stress, acute stress, multifactorial stress etc.) in developing animal models of IBS, which also is the case of this study.

A few recent studies indicate that oxidative stress may also be a contributing factor in the pathogenesis of IBS [23, 24]. Thus, it is noticed in numerous metabolic disorders that under inflammatory circumstances, immune cell activation leads to reactive oxygen species (ROS) generation at the site of inflammation, with a potential subsequent mutual amplification of the two processes [25–29].

Furthermore, intestinal microbiota, commensal and pathogenic, particularly under dysbiotic circumstances, contributes to the generation of ROS by stimulating the cellular response of phagocytic cells (neutrophils, macrophages) as well as of other cell types, including intestinal epithelia, in response to microbial signals and metabolic products [30].

Moreover, clinical and experimental studies concerning oxidative stress IBS brought consistent evidence that nitrosative and oxidative products could also contribute to IBS pathogenesis. In this way, human clinical studies showed increased nitric oxide synthase (NOS) activity and increased nitric oxide levels [31] which together with decreased antioxidant capacity could lead to increased lipid peroxidation as shown by [23]. Regarding antioxidant enzymes, decreased superoxide dismutase, catalase, and glutathione peroxidase activities were described for IBS patients, as compared to the healthy controls [32, 33]. These observations were also confirmed by IBS animal model studies of oxidative stress implication description as significantly increased lipid peroxidation and reduced antioxidant defense in intestinal tissues of several IBS rat models [5, 34].

Currently, no curing treatment for IBS has been identified, and management of the disorder is aimed at relieving the different symptoms, using various pharmaceutical drugs, such as loperamide, probiotics, anticholinergics and antispasmodics, tricyclic antidepressants, antibiotics, or psychological therapies—cognitive behavioral therapy, hypnotherapy etc. [35–38]. Curing the disorder should rather be targeted towards a more inclusive and personalized lifestyle improvement [35]. In seeking valid solutions for a more encompassing approach, natural herbs and plants can have the potential to provide new compounds of therapeutical agents, without side effects.

*Chrysanthellum americanum* L. Vatke is a small erect or less prone herbaceous plant with very few leaves and yellow flowers belonging to the *Asteraceae* family, commonly found throughout tropical Africa and America [39]. This aromatic plant is used in Burkina Faso and Central African traditional medicine in the treatment of fever, hepatitis, jaundice, and dysentery [40] but is also well-known in herbal medicine research area. The bioactive compounds identified in the *C. americanum* extract are known to possess antioxidant, P-vitamin, and antilithiasis remarkable properties [40, 41]. Most of the therapeutical properties of *C. americanum* extracts are attributed to the saponins (chrysanthellin A and B) and flavonoids (luteolin 7-O-glucoside, eriodictyol 7-O-glucoside, isookanin 7-O-glucoside or flavonomarein, okanin 4′-O-glucoside or marein, and maritimetin 6-O-glucoside or maritimein) [42]. The psychoactive principles found in the methanolic extract from the plant were referred to be able to cross the blood-brain barrier and have potential in the treatment of epilepsy and psychosis [43–45].

*In vitro* assay of crude and polyphenolic *C. americanum* extract effects on liver tissue studies showed lipid peroxidation inhibitory activities similar to quercetin, a commonly used antioxidative standard in biochemical determinations [46]. Hence, considering the antioxidative properties and the protective effects on the nervous system [47] and its traditional use for good digestion, we decided to investigate the effect of a hydroethanolic extract of *C. americanum* on the memory processes, anxiety, and depressive-like behavior of a rat model of chronic stress-induced IBS and the influence on the brain oxidative stress marker dynamics.

## 2. Materials and Methods

### 2.1. Plant Material and Extraction

*Chrysanthellum americanum* L. Vatke whole plants were collected during August 2014 in Loumlila, 15 km north of Ouagadougou, capital of Burkina Faso. The plant was identified by Prof. Millogo-Rasolodimby from the Vegetal Biology Department of the University of Ouagadougou. A voucher specimen (ID-10474) was deposited at the Herbarium of the Laboratory of Vegetable Biology and Ecology of the University of Ouagadougou.

The whole plant was dried at room temperature and ground to fine powder. Seventy-five gram of this powder was macerated during 48 hours with mechanical stirring using 750 mL of aqueous ethanol (80% *v*/*v*) in laboratory conditions. Following this, extract solutions were concentrated under reduced pressure in a rotary evaporator (BÜCHI, Rotavapor R-200, Switzerland) at approximately 40°C, frozen and lyophilized using a lyophilizer (Telstar-Cryodos 50, Spain). The obtained aqueous ethanol extracts (crude extract) were fractionated in solvents of increasing polarity (dichloromethane, ethyl acetate, butanol, and water residuals). Butanol fraction (polyphenol extract) was dried and weighted before packed in waterproof plastic flasks and stored at 4°C until use. The yields of crude aqueous ethanol extract and polyphenol extract were 8.00% and 6.22%, respectively.

Dry polyphenolic extract dissolved in physiological saline (0.9 mg/mL) was used for animal treatments.

### 2.2. Animals

Twenty-four female white Wistar rats weighing 200 ± 18.70 g at the start of the experiment were used. The animals were housed in a temperature and light-controlled room (20°C, 55–60% relative humidity and light-dark cycle of 12 h cycle starting at 08:00 AM). The animals were habituated for one week following their arrival before the stress paradigm was initiated. Food and water were provided *ad libitum* except for two 24-hour periods when food or water deprivation stress was applied. Rats were treated in accordance with the guidelines of animal bioethics from the Act on Animal Experimentation and Animal Health and Welfare Act from Romania, and all procedures were in compliance with Directive 2010/63/EU of the European Parliament and of the Council of 22 September 2010 on the protection of animals used for scientific purposes.

### 2.3. Chemicals

To carry out our activities, we used analytical grade solvents and various classic reagents. Ethyl acetate and 2-thiobarbituric acid were purchased from Sigma-Aldrich (Steinheim, Germany); potassium persulfate, 2,2′ azino-bis(3-ethylbenzothiazoline 6-sulphonate), and trichloroacetic acid were supplied by Fluka Chemie (Buchs, Switzerland); dichloromethane, ferric dichloride, and ethanol were sourced from Probalo (Paris, France); butanol was sourced from SDS (Peyin, France). For cerebral tissue oxidative stress biomarkers, we used the spectrophotometric SOD Assay Kit (Fluka Chemie), GPX cellular activity assay kit CGP-1 (Sigma Chemicals), trichloroacetic acid 50%, and thiobarbituric acid 0.73%.

### 2.4. *In Vivo* Experiments

#### 2.4.1. Irritable Bowel Syndrome Induction

In order to induce the IBS symptoms to rats, we used a combined multifactorial heterotypic chronic and subchronic stress-exposure paradigm, as described with some variations by previous studies in the literature [48, 49]. For the current experiment, we adapted the method by using an original combination of stressors in order to optimize the protocol and reduce the time of exposure, which we presented previously [50]: thus, the IBS groups were exposed each day to two stress factors for a relative short period of only seven days. First stressor was constantly used each morning and succeeded by a second stress factor that differed daily in order to minimize habituation. IBS-modeled rats were exposed to one-hour water avoidance stress during the first half of the day, for the entire week; the standard procedure consists in placing the rat on a small platform (8 × 6 cm) in the middle of a small plastic basin filled with warm water (25°C) at the height level of the platform [51]. Control group rats were placed on the same platform but in a waterless container for 1 hour. Six hours after the water avoidance stress paradigm, the IBS group was exposed to one of the six different stressors for each of the last six days of the protocol: (1) predator sound exposure for 5 minutes, (2) water deprivation for 24 hours, (3) imitation of an abdominal injection, (4) tilted cage in a 45° angle for 12 h, (5) painful tailpinch with a clothespin for two minutes, and (6) food deprivation for 24 hours [48]. Behavioral tests were conducted between the interval 10:00 AM and 16:00 PM, following the stress exposure period, the extract administration period and one additional day for rest. The administration of the polyphenolic extract of *C. americanum* was carried out by oral gavage for five consecutive days, beginning in the last two days of the stress exposure protocol and continuing three days afterwards.

#### 2.4.2. Experimental Design

The rats were divided into four groups (*n* = 6 animals per group): (1) the control group received physiological saline (2.5 mL/kg body weight) consecutively for six days; (2) the extract-treated group received *C. americanum* polyphenolic extract (100 mg/kg body weight) for six days consecutively; (3) the IBS group received physiological saline (2.5 mL/kg body weight) two days during and four days after rats were exposed to multifactorial stress paradigm; (4) the IBS-extract (IBS-E group) group received *C. americanum* polyphenolic extract (100 mg/kg body weight) two days during and four days after rats were exposed to multifactorial stress paradigm.

Physiological saline (0.9 mg/mL) and dry polyphenolic extract dissolved in physiological saline were used, and all treatments were carried out using intragastrically administration by oral gavage on an empty stomach, one hour before any stress exposure paradigm.

#### 2.4.3. Behavioral Assessment

*2.4.3.1. Y-Maze Task*. For assessing short-term memory modifications, we used the reliable Y-maze task, as described in a previous study [52]. The spontaneous alternation percentage, defined by the consecutive entries into all three arms of the maze, was analyzed as the behavioral marker to reflect spatial working memory.

*2.4.3.2. Elevated Plus Maze Task (EPM)*. Anxiety-like behavior was assessed in the EPM paradigm which was also described in our previous study from 2018 [52]. Rats were placed at the juncture of the open and closed arms, and the amount of time spent on the open arms was recorded during a 5 min test. Besides time spent in the open arms, head-dipping behavior, time of grooming, and periods of freezing were recorded for assessing anxiety.

*2.4.3.3. Forced Swimming Test (FST)*. The antidepressant effects of the polyphenolic extract of *C. americanum* on rats with IBS were assessed using the method described by [53], but with some modifications: rats were individually placed into transparent glass cylinders (diameter 30 cm, height 59 cm) containing 25 cm of water at 24 ± 1°C and were let to swim for a 6-minute period. During the last 4 minutes of the swimming session, the following behavioral responses, relevant for depressive behavior, were recorded: (1) immobility (time spent floating with the minimal movements to keep the head above the water) and (2) swimming (time spent with active swimming movements).

#### 2.4.4. Sample Preparation

Following 24 hours after behavioral tests, rats were anesthetized (ketamine 100 mg/kg, xylazine 10 mg/kg) and rapidly decapitated. The temporal lobes were collected, weighed, and homogenized with a Potter Homogenizer coupled with Cole-Parmer Servodyne Mixer in bidistilled water (1 g tissue/10 mL bidistilled water). The samples were centrifuged for 15 min at 3000 rpm; the supernatant was separated and pipetted into tubes.

#### 2.4.5. Biochemical Determinations

*2.4.5.1. Superoxide Dismutase Determination*. For determining SOD enzymatic activity, we used a spectrophotometric SOD Assay Kit according to the manufacturer's instructions as described in a previous study [52]. The method is based on the water-soluble tetrazolium salt reaction with superoxide anion producing a water-soluble formazan dye. On this substrate, the O_2_ reduction rate linearly related to xanthine oxidase activity and inhibited by SOD is an indirect way of colorimetric determination. The assay endpoints were monitored by absorbance at 450 nm after 20 min of reaction time at 37°C. The inhibition rate (%) was then reported considering the total protein content from the sample. The SOD activity was therefore expressed as enzyme units/milligram (U/mg).

*2.4.5.2. Glutathione Peroxidase (GPx) Determination*. For the determination of GPx activity, we used an indirect assay method, previously described [54] based on a GPx cellular activity assay kit CGP-1 (Sigma Chemicals), that measures the rate of NADPH consumption (340 nm) during the considered time unit for the GPx-catalyzed oxidation of glutathione (GSH) to oxidized GSSG which is then coupled with recycling GSSG back to GSH utilizing glutathione reductase (GR) and NADPH. The decrease in NADPH at 340 nm during oxidation of NADPH to NADP is indicative of GPx activity (anis). The results were reported to the total protein sample content, and GPX activity was expressed as GPx enzyme units/mg (U/mg).

*2.4.5.3. Malondialdehyde Determination*. Malondialdehyde (MDA) concentrations were determined using the thiobarbituric acid reactive substance (TBAR) assay method, previously described [54]. 200 *μ*L of supernatant was added and mixed with 1 mL of trichloroacetic acid at 50%, 0.9 mL of TRIS-HCl (pH 7.4), and 1 mL of thiobarbituric acid at 0.73%. After vortex mixing, samples were maintained at 100°C for 20 min. The samples were then centrifuged (3000 rpm, 10 minutes), and the supernatant was read at 532 nm in a UV-VIS spectrophotometrical system (Beckman Coulter, Canada). The signal was read against an MDA standard curve, and the results were expressed as nmol/mg protein.

## 3. Statistical Analysis

Results were statistically analyzed using one-way analysis of variance (ANoVA) by using SPSS. All of the results are expressed as mean ± SEM. Tukey's test was used to determine the level of significance of all results obtained on XLSTAT 7.1. Results were regarded as significant at *p* < 0.05.

## 4. Results

### 4.1. Effect of *Chrysanthellum americanum* Polyphenolic Extract on Short-Term Memory in Y-Maze Task

**Figure 1:**
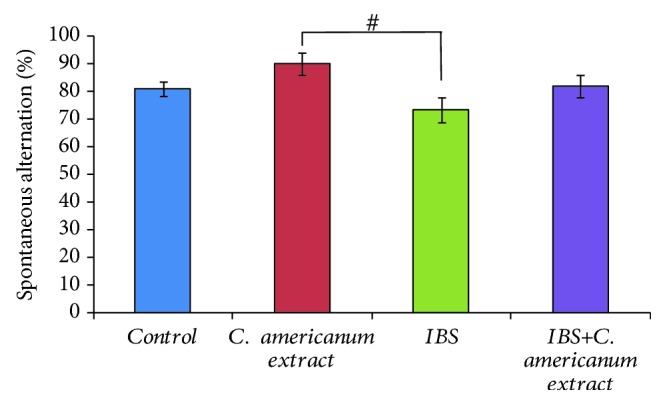
*C. americanum* extract treatment effects on short-term memory in the extract group and chronic stress-exposed rats (IBS-E group) in the Y-maze test expressed as spontaneous alternation (%). The values are expressed as means ± S.E.M. (*n* = 6 per group; ^#^*p* = 0.03 for the *C. americanum* extract-treated group vs. the IBS group).

*C. americanum* extract effect on short-term memory within Y-maze task is shown in Figure 1. Analysis of the spontaneous alternation percentage showed a statistically significant difference between all groups of rats (*F*(3, 12) = 5.11, *p* = 0.009). The decrease in the spontaneous alternation percentages observed in the stress-exposed rats (IBS groups) was not statistically significant vs. the control group (*F*(1, 9) = 5.73, *p* = 0.31) but was significantly lower than the extract-alone-treated rats (*F*(1, 9) = 5.73, *p* < 0,034). However, this significant decrease was not observed in the case of the IBS-extract group, which did not differ significantly from the control or extract-alone groups.

### 4.2. Effect of *Chrysanthellum americanum* Polyphenolic Extract on Anxiety Behavior in the Elevated Plus Maze Task

**Figure 2:**
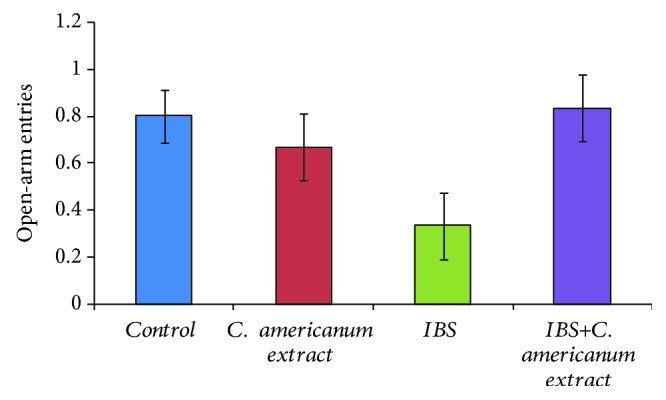
*C. americanum* extract treatment effects on the number of open-arm entries made in the elevated plus maze. The values are expressed as means ± S.E.M. (*n* = 6 per group).

**Figure 3:**
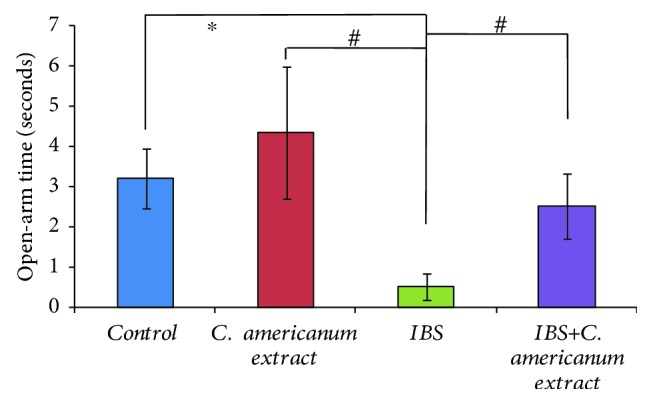
*C. americanum* extract treatment effects on the time spent in the open arms in the elevated plus maze. The values are expressed as means ± S.E.M. (*n* = 6 per group; ^∗^*p* = 0.02 for the IBS group vs. the control group; ^#^*p* = 0.04 for the *C. americanum* extract-treated group and the IBS *C. americanum* extract-treated group vs. the IBS group).

In the elevated plus maze task, the administration of the *C. americanum* extract showed to have anxiolytic effects by increasing the number of open-arm entries of IBS-extract-treated rats as compared to the IBS stress-exposed group (*F*(1, 10) = 3.46, *p* = 0.09), to similar values as those of the control and the extract-alone groups (Figure 2). The absence of clear statistical difference may be explained by the general tendency of all individuals to make no more than 1 open-arm entries, except for the IBS group, that generally avoided open-arm exploration. Moreover, these results were further confirmed by analyzing the time spent in the open arms (Figure 3). The ANoVA statistical test revealed that treatment with *C. americanum* extract significantly increased the time spent in the open arms in the extract-treated group, as compared to the IBS stress-exposed group (*F*(1, 10) = 5.18, *p* < 0.04) and also in the IBS-extract-treated group, as compared to the IBS stress-exposed group (*F*(1, 10) = 5.21, *p* < 0.04). Significantly decreased time spent in the open arms when compared to the control group also suggested high anxiety status in the IBS group (*F*(1, 10) = 8.81, *p* < 0.02).

**Figure 4:**
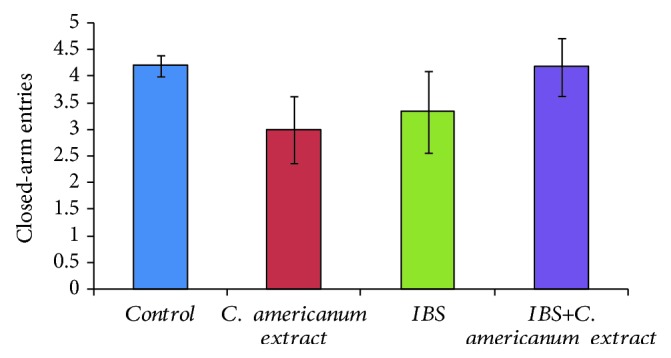
*C. americanum* extract treatment effects on the number of closed-arm entries in the elevated plus maze. The values are expressed as means ± S.E.M. (*n* = 6 per group).

However, regarding exploratory activity, ANoVA showed no significant difference between studied groups on the number of crossings from one closed arm to the other (Figure 4).

### 4.3. Antidepressant Effects of *Chrysanthellum americanum* Polyphenolic Extract in the Forced Swimming Test

**Figure 5:**
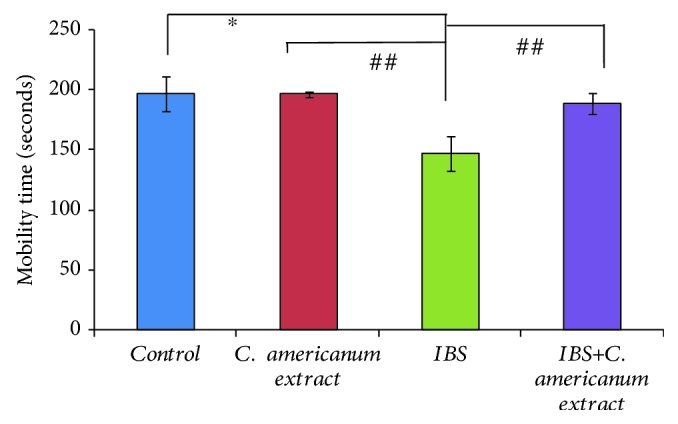
Effects of *C. americanum* extract treatment after stress exposure on swimming time in the forced swimming test. The values are expressed as means ± S.E.M. (*n* = 6 per group; ^∗^*p* = 0.01 for the IBS group vs. the control group; ^##^*p* < 0.01 for the *C. americanum* extract-treated group and the IBS+*C. americanum* extract-treated group vs. the IBS group).

**Figure 6:**
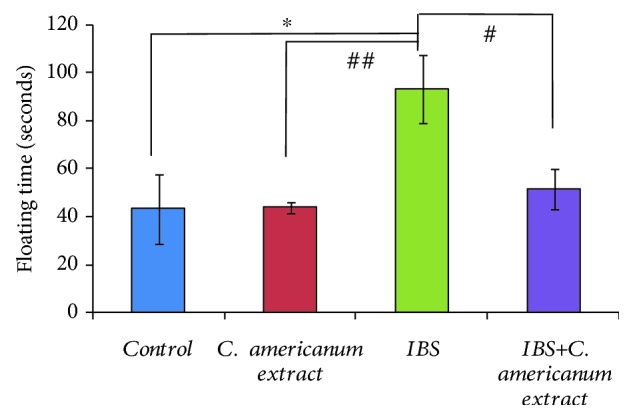
Effects of *C. americanum* extract treatment after stress exposure on immobility time in the forced swimming test. The values are expressed as means ± S.E.M. (*n* = 6 per group; ^∗^*p* = 0.01 for the IBS+*C. americanum* extract-treated group vs. the IBS group; ^#^*p* ≤ 0.05 for the IBS group vs. the control group; ^##^*p* < 0.01 for the *C. americanum* extract-treated group vs. the IBS group).

Evaluation of antidepressant effects of the *C. americanum* extract by FST highlighted statistically significant differences between groups after one-way ANoVA in both terms of swimming time (*F*(3, 20) = 5.86, *p* < 0.0048) (Figure 5) and on the immobility time (*F*(3, 20) = 5.55, *p* < 0.0061) (Figure 6). While repeated stress exposure clearly caused depressive-like behavior in the IBS group: significantly decreased swimming time (*p* = 0.01) (Figure 5) and increased immobility (*p* = 0.035) (Figure 6) vs. the control group, treatment with *C. americanum* extract showed a potent antidepressive effect, for both treated groups (extract and IBS-extract groups). Most importantly, this beneficial reversing effect stands out in the IBS-extract-treated group, suggested by the significant increase in swimming time (*p* = 0.03), as compared to the IBS group (Figure 5) and reduction of immobility period (*p* = 0.031992), as compared to the stress-exposed IBS group (Figure 6).

### 4.4. Biochemical Assay of Oxidative Stress Biomarkers

#### 4.4.1. GPx Activity Assay

**Figure 7:**
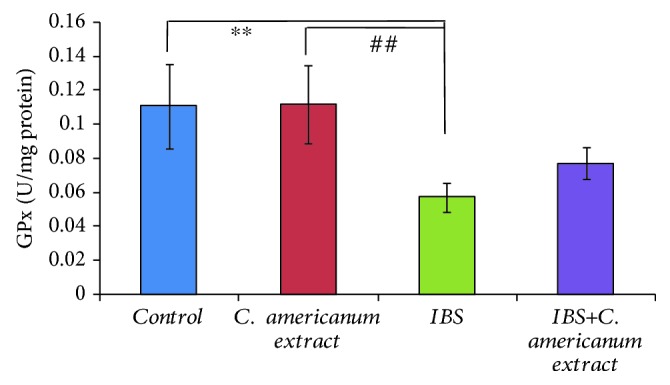
The effects of *C. americanum* extract treatment after stress exposure on GPx activity in the temporal lobe. The values are expressed as means ± S.E.M. (*n* = 6 for each group; overall *p* = 0.001; ^∗∗^*p* < 0.01 for the IBS group vs. the control group; ^##^*p* < 0.01 for the *C. americanum* extract-treated group vs. the IBS group).

Following the stress exposure protocol, GPx enzymatic activity measured in the temporal lobes registered a significant decrease in the IBS group, as compared to the control group (*F*(1, 11) = 12.93, *p* = 0.0042). Similarly, a significant difference was observed between the extract-treated and IBS groups (*F*(1, 11) = 15.69, *p* = 0.0022). No statistically significant differences were observed between the IBS and extract-treated IBS groups; however, the latter did not differ significantly from the unstressed groups, which is suggestive for an improvement in the antioxidative activity (Figure 7).

#### 4.4.2. SOD Activity Assay

**Figure 8:**
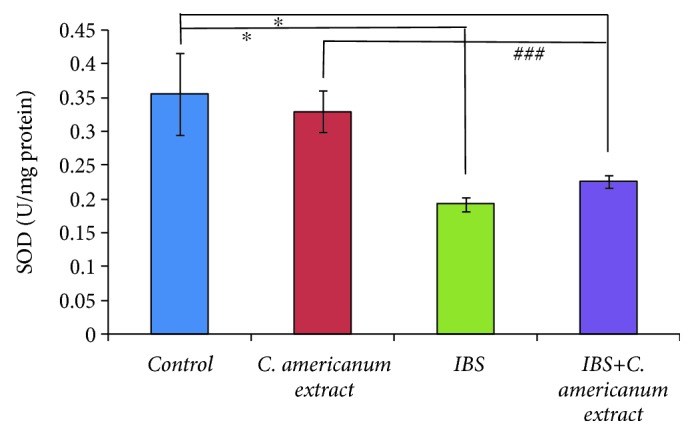
The effects of *C. americanum* extract treatment after stress exposure on SOD activity in the temporal lobe. The values are expressed as means ± S.E.M. (*n* = 6 for each group; overall *p* = 0.001, ^∗^*p* < 0.05 for the IBS group and the IBS+*C. americanum* extract-treated group vs. the control group; ^###^*p* < 0.01 for the *C. americanum* extract-treated group vs. the IBS and IBS+*C. americanum* extract-treated groups).

Significant overall differences between groups for SOD activity in the mice temporal homogenates were observed (*F*(3, 25) = 6.83, *p* = 0.0016) (Figure 8). As in the case of GPx enzymatic activity, we obtained a significant decrease in SOD activity for the IBS group as compared to the control group (*F*(1, 11) = 8.73, *p* = 0.013) and to the extract-treated group (*F*(1, 14) = 20.46, *p* = 0.00047) (Figure 8). Also, SOD activity was significantly decreased in the stress-exposed extract-treated group, as compared to the control group (*F*(1, 11) = 5.34, *p* = 0.04) and the extract-treated group (*F*(1, 13) = 10.44, *p* = 0.006) (Figure 8).

#### 4.4.3. MDA Assay

**Figure 9:**
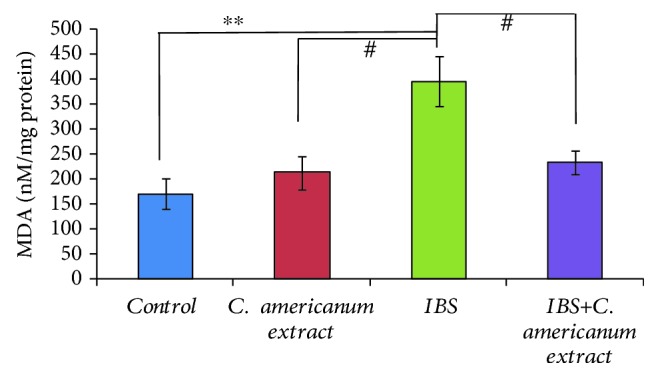
The effects of *C. americanum* extract treatment after stress exposure on MDA concentrations in the temporal lobe. The values are expressed as means ± S.E.M. (*n* = 6 for each group; overall *p* = 0.001; ^∗∗^*p* < 0.01 for the stress-exposed IBS group vs. the control group; ^#^*p* < 0.05 for the *C. americanum* extract-treated group and the IBS+*C. americanum* extract-treated group vs. the IBS group).

A distinct increase in the temporal lobe MDA concentrations, a lipid peroxidation marker, was observed in the stress-exposed IBS group, as compared to all other groups: *p* = 0.003 vs. the control group, *p* = 0.011 vs. the extract-treated group, and *p* = 0.014 vs. the stress-exposed extract-treated group (Figure 9).

### 4.5. Pearson's Correlation and Regression Analysis

**Figure 10:**
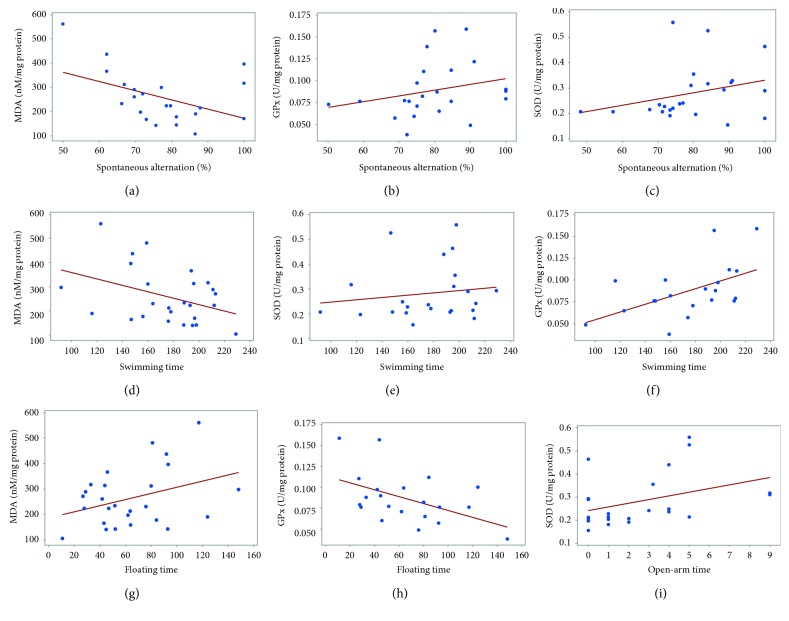
Pearson's correlations between spontaneous alternations (%) vs. MDA (a), GPx (b), SOD (c), swimming time vs. MDA (d), vs. SOD (e), vs. GPx (f), immobility (floating) time vs. MDA (g), vs. GPx (h), and open-arm time vs. SOD (i).

Pearson's correlation coefficient and regression analysis were performed in order to analyze the connection between oxidative stress biomarkers and behavioral results. Interestingly, significant correlations were found between the spontaneous alternation (%) vs. MDA (*r* = −0.449, *p* < 0.036) (Figure 10(a)) and to a lesser extent vs. GPx (*r* = 0.228, *p* < 0.205) (Figure 10(b)) or SOD activities (*r* = 0.259, *p* < 0.245) (Figure 10(c)). However, strong correlations were evidenced by linear regression between swimming time in FST vs. MDA (*r* = −0.384, *p* < 0.05) (Figure 10(d)), vs. SOD (*r* = 0.143, *p* < 0.05) (Figure 10(e)), and GPx activities (*r* = 0.528, *p* < 0.01) (Figure 10(f)) and in the same FST task, between immobility time vs. MDA (*r* = 0.354, *p* < 0.05) (Figure 10(g)) and vs. GPx activity (*r* = −0.487, *p* < 0.05) (Figure 10(h)). Also, a significant correlation was observed between time spent in the open arms of the EPM vs. SOD specific activity (*r* = 0.386, *p* < 0.05) (Figure 10(i)).

## 5. Discussion

The present data indicate that our butanolic extract of *C. americanum* has protective effects on the nervous system as it significantly decreases brain oxidative stress levels and anxiety and depressive-like behavioral manifestations in chronic stress-induced IBS mice.

Our recent results showed low antioxidant capacity in total enzymatic antioxidant capacity (TEAC) but high capacity in reducing lipid peroxidation; also, the butanol fraction is richer in total phenolic compounds and in flavonoids, as compared to the crude extract [55].

It was previously shown that *Chrysanthellum* species extracts have high antioxidant capacity but also would possess positive impact on the central and peripheral nervous system [47, 56].

Regarding cognitive performance in the Y maze task, when administered to IBS rats, the extract showed a facilitatory effect on short-term memory. A previous report indicates that stress induced by IBS has a negative impact on working memory [57]. However, IBS patient studies on potential cognitive deficits bring contradictory results and do not find convincing associations between IBS and cognitive/memory impairment *per se* [58, 59]. Thus, dysfunctional cognition is reported in clinical studies but as a consequence of concurrent mood comorbidities or of the whole psychological burden than as an intrinsic process [60–62].

In our present study, the decreased memory performance observed in the IBS group was not statistically significant. Thus, certain cognitive impairments were noticeable in the current IBS model by indirect assessment, as a significant difference occurred between stress-exposed rats and solely extract-treated rats.

Previous studies showed that failure of stress-coping mechanisms in some IBS subsets patients could lead to inadequate neuroendocrine autonomic responses along the HPA, such as altered cortisol levels and increased vagal activation [8, 63, 64]. In this way, their sensory information processing is impaired by the overload of the emotional circuitry, as shown by some sparse studies on functional magnetic resonance imaging in IBS subjects [65] or immunochemistry in animal models [66]. Even more interesting, persistent stress in animal models is shown to morphologically alter hippocampal circuitry and suppress neurogenesis [8], while two recent studies of Mayer and colleagues demonstrate decreased gray matter density in prefrontal cortex, posterior parietal cortex, pregenual anterior cingulate cortex, and similar trends in the posterior insula/secondary somatosensory cortex, (para)hippocampus, regions involved in cognitive functions among others [67]. Also, several correlations between gut microbiota status and brain structural alterations in certain gut microbiome-based IBS subgroups of patients [68] were established. Regarding mood behavior, results for the IBS rat groups were statistically significantly associated with depression in the FST test and anxiety in EPM, in line with the widely literature reported data in both human patients [58, 69] and animal models [70].

The dysfunctional gut-brain axis pathways encompassing stress-related alterations along the HPA axis (and dysbiosis) are vastly reported to be linked with dysbiotic systemic inflammatory phenomena [71–73], which are speculated to trigger neuroinflammation in brain regions including the hippocampus and cerebellum [74, 75]. Numerous studies particularly in chronic neurodegenerative diseases highlighted the interrelation between the neuroinflammatory abnormal cytokine production and activation of immune cells and increased release of prooxidants in a reciprocal manner [76–79].

We also reported here dysfunctionalities in the antioxidant enzymatic systems. Inappropriate antioxidant capacity plays an important role in altering the hippocampal-dependent memory process [80–82]. In SOD-deficient mice, Huang et al. [83] observed that diminished hippocampal neurogenesis and altered dendritic structure accompanying the compromised oxidative system resulted in reduced memory capacity. Significantly upregulated antioxidant enzymatic genic expression of SOD and GPx (to counter the increased levels of ROS) and increased lipid peroxidation in the hippocampus of an epileptic rat model were associated with high number of memory errors in the radial arm maze [84]. However, there are many reports in clinical and statistical studies that IBS symptoms and mood disorders, especially depression and anxiety, are correlated reciprocally to perpetuate a cycle of anticipatory worries and autonomic gastrointestinal disruptions [85–89].

Again, maladaptative reaction to stressful life episodes increases vulnerability to psychopathological phenomena through several mechanisms which include abnormal secretion of CRF and immunomodulating glucocorticoids and also dysbiosis. Gastrointestinal autoimmune and inflammatory reactions (gut hyperpermeability and neuroendocrine factors translocation) are suggested to modulate oxidative stress status in certain brain areas, including the hippocampus and amygdala, involved in the development of depression and anxiety [90, 91]. In this way, Bouayed et al. [92] reported significantly high levels of lymphocytic, granulocytic, and monocytic intracellular ROS in mice with high anxiety traits. Moreover, it seems that oxidative stress mechanism components, such as glutathione reductase 1 and glyoxalase 1, are directly involved in anxiety-like behavior modulation, as Hovatta and colab. firstly showed [93].

Clinical studies also report correlations between lowering of antioxidative status determined by measuring the redox potential of urine and higher anxiety and depression state values in normal individuals [94] and also significantly higher total oxidant status and oxidative stress index values in IBS patients when compared to control [24].

Several studies bring evidence that mood disorders are accompanied by oxidative stress reflected by increased xanthine oxidase activity in the cortico-limbic-thalamic-striatal regions in post-mortem brain tissue of patients with recurrent depressive episodes [95], by increased levels of plasma malondialdehyde [96, 97].

Phytochemicals and polyphenols are well known as efficient free radical scavengers, and recent studies suggest their potential in reversing oxidative stress and improving mood and cognitive psychopathologies [84, 98, 99].

The flavonoids, saponides, tannins, and various essential oil terpene-rich *C. americanum* plant medicinal potential is recognized due to its long-time use in Western African traditional medicine [35, 100]. The main saponin-based components of *C. americanum*, chrysanthellin A and B, were suggested to be useful in the therapy of digestive impairments [101, 102], but there is no study up to date to investigate any specific neuroprotective or antioxidant effects.

However, it was clear to suggest that *C. americanum* extracts could possess neurocognitive activity while Mévy and colab. [40] showed that several of their compounds are capable to cross the blood-brain barrier. Also, tyrosine and phenylalanine contained by the extract could assist dopamine and adrenaline syntheses [42, 103]. Moreover, flavonoid contents could exert antidepressive and anxiolytic effects [104, 105]. In this study, we are furtherly explaining the potential of *C. americanum* extract potential to alleviate psychocognitive deficiencies in relation to its antioxidant capacity for the first time in our best knowledge.

Regarding the limitations of our study, we must mention the used animal model validation sequel, as we based it on previous protocols [43, 44], which we adapted for stress effect increase. Thus, the visceral hypersensitivity was not assessed in this study. Brief intestinal transit and stool change assessment were carried out and highlighted a slight reduction in the number of faecal boluses in the IBS group. However, Ghédira and Goetz [100] described a potential laxative side effect of *C. americanum* integration.

In our study, we found that the administration of the polyphenolic extract significantly decreased the oxidative burden in the temporal lobe, particularly by decreasing the MDA levels and improving GPx activity. In this way, this could suggest that the flavonoids which could be found in the extract are responsible for antioxidant activity, while they are recognized to scavenge ROS and chelating metal ions [106]. Thus, luteolin, a polyphenolic flavon compound present in the extract as luteolin 7-O-glucoside, has been recently found to protect against sodium nitroprusside-induced oxidative damage in mouse brain and against Fe^(2+)^-induced lipid peroxidation in mouse brain homogenate [107] and also to significantly inhibit superoxide anion generation and ROS production and diminish neutrophil inflammatory responses in the mice model of inflammatory arthritis [108].

Also described as a *C. americanum* extract component, okanin 4′-O-glucoside was noted for its neuroprotective effect in diabetic encephalopathy, by attenuating methylglyoxal-induced damages in the mitochondrial function and production of ROS, and increasing glyoxalase I enzymatic activity [109], key enzyme in cellular defense against glycative and oxidative stress [110]. Eriodictyol, present in the extract as eriodictyol 7-O-glucoside, is also known as a potent flavonoid with a higher antioxidant capacity than quercetin, luteolin, or taxifolin [111]; while eriodictyol increased the activity of enzymatic and nonenzymatic antioxidants in the circulatory system, liver, and colon, it is significantly decreasing mucosa and faecal bacterial enzyme activities [112].

## 6. Conclusion

*Chrysanthellum americanum* polyphenolic extract use in a stress-induced animal model of irritable bowel syndrome, a condition influenced by oxidative stress and which has a negative impact on the patient psychology, resulted in a slight memory-enhancement effect and a significant decrease of anxiety and depressive-like behavior. The assessments of oxidative stress markers showed a significant decrease of the superoxide dismutase and glutathione peroxidase-specific activities in the stress-exposed IBS animal model temporal lobes. Short-term administration of *Chrysanthellum americanum* extract significantly attenuated the increased malondialdehyde levels and glutathione peroxidase-specific activity. Significant correlations were identified by Pearson linear regression analysis between oxidative stress biomarkers and behavioral indicators. Therefore, our results suggest that administration of *Chrysanthellum americanum* polyphenolic extract can alleviate mood and cognitive impairments related to stress-induced IBS condition by attenuation of the cerebral oxidative stress.

## Data Availability

The data regarding the behavioral tasks and the oxidative stress markers used to support the findings of this study are included within the article (correlations and individual presentation of data before that).
